# *Mycobacterium riyadhense* Infection Presenting as a Central Nervous System Mass: A Case Report

**DOI:** 10.1155/crdi/2820094

**Published:** 2026-05-26

**Authors:** Ibrahim Bahabri, Rahmah S. Alzahrani, Nouf Althabit, Musaad A. Alhurayyis, Salim Baharoon, Ahmad Alharbi

**Affiliations:** ^1^ Department of Medicine, King Abdulaziz Medical City, Ministry of National Guard-Health Affairs, Riyadh, Saudi Arabia, ngha.med.sa; ^2^ Internal Medicine Department, Infectious Diseases Division, King Abdulaziz Medical City, Ministry of National Guard-Health Affairs, Riyadh, Saudi Arabia, ngha.med.sa; ^3^ Radiodiagnostics & Medical Imaging Department, Prince Sultan Military Medical City, Riyadh, Saudi Arabia, psmmc.med.sa; ^4^ Department of Critical Care, King Abdulaziz Medical City, Ministry of National Guard-Health Affairs, Riyadh, Saudi Arabia, ngha.med.sa; ^5^ College of Medicine, King Saud bin Abdulaziz University for Health Sciences, Riyadh, Saudi Arabia, ksau-hs.edu.sa; ^6^ King Abdullah International Medical Research Center, Riyadh, Saudi Arabia, kaimrc.med.sa

## Abstract

*Mycobacterium riyadhense* (*M. riyadhense*) is a slow‐growing nontuberculous mycobacterium that is increasingly being recognized as a cause of pulmonary and extrapulmonary disease, primarily in Saudi Arabia. Here, we report a case of *M. riyadhense* with an unusual presentation as a central nervous system mass, with an initial diagnosis of neurosarcoidosis and notable improvement with the use of steroids and rituximab prior to establishing the final diagnosis.

## 1. Introduction

Nontuberculous mycobacteria (NTM) are a diverse group of bacteria with a wide array of species found in numerous environmental sources and animal reservoirs [1]. These bacteria are capable of causing pulmonary and extrapulmonary disease in immunocompetent and immunocompromised hosts; the most commonly affected system is the pulmonary system, followed by the lymphatic system, with other reported cases involving the central nervous system (CNS) [2–7]. Recognition of NTM species in clinical settings has become more feasible with the refinement of molecular technologies, such as matrix‐assisted laser desorption ionization‐time‐of‐flight mass spectrometry (MALDI‐TOF MS) platforms [8].

*Mycobacterium riyadhense* (*M. riyadhense*) is a slow‐growing NTM first isolated as a cause of sinusitis in a young immunocompetent patient [9]. It has subsequently been isolated as a pulmonary pathogen at multiple sites worldwide, including France, Bahrain, Italy, Germany, and South Korea [10, 11]. To date, there are only two case reports of CNS infections by *M. riyadhense* [12]. Here, we present a case of *M. riyadhense* infection presenting as an intradural mass, with an undulating journey through diagnosis and treatment.

## 2. Case Presentation

The patient was a 29‐year‐old woman with a history of mild asthma and prior caesarean section; she presented with an 18‐month history of neck pain, radiating to her trunk, associated with descending numbness and weakness in all 4 limbs, worse on the left side. Two months prior to presenting to the ER, she developed urinary urgency with occasional urge incontinence. A review of systems revealed oropharyngeal dysphagia, exertional dyspnea, and left lower limb pain and swelling. There was no history of constitutional symptoms, cough, or hemoptysis. She had no sick contacts, no pets at home, no contact with farm animals, no raw milk ingestion, and no history of travel outside the country.

Her physical examination revealed decreased strength in both upper limbs (4/5) and both lower limbs (4/5 on the right, 3/5 on the left), with impaired fine touch and pressure on the left side, starting at the T4 level and below. Gait examination revealed limping on her left side. Spurling’s test was positive on the left side, and Hoffman’s sign was positive bilaterally. Babinski’s test was positive in both lower limbs, more prominent on the left side.

The initial laboratory results obtained at our hospital were as follows: total white blood cell (WBC) count of 7.8 × 10^9^/L with a normal differential (normal range: 4–11 × 10^9^/L), hemoglobin level of 109 gm/L (normal range: 120–160 gm/L), platelet count of 507 × 10^9^/L (normal range: 150–400 × 10^9^/L), erythrocyte sedimentation rate (ESR) of 67 mm/hour (normal range: 0–30 mm/hour), and C‐reactive protein (CRP) of 12 mg/mL (normal reference value: ∼5/mg/L). The serological results for *Brucella* species (*abortus and melitensis*) were negative, and the syphilis screening (total antibody) and human immunodeficiency virus (HIV) screening (4th generation antigen/antibody assay) results were both negative.

Computed tomography (CT) of the cervical spine revealed an enhancing soft tissue mass occupying the spinal canal opposite C2 and extending downward to the C7 vertebra, with diffuse bone erosion and sclerosis. MRI of the cervical spine further characterized the mass as an intraspinal epidural, paraspinal, avidly enhancing cervical lesion with multiple neural foraminal extensions and cord compression, which was most severe at C5 (Figure 1). The impression of the radiographic appearance was suggestive of a neoplastic process, with the most likely malignancies based on the location being diffuse large B‐cell lymphoma (DLBCL), as well as Hodgkin and non‐Hodgkin lymphomas; other less likely differential diagnoses included tuberculosis (TB) and brucellosis. Imaging of the brain and the rest of the spine was unremarkable, a lower limb ultrasound with Doppler showed left distal femoral vein thrombosis, and a chest and abdomen CT scan for staging of suspected lymphoma was unremarkable.

**FIGURE 1:**
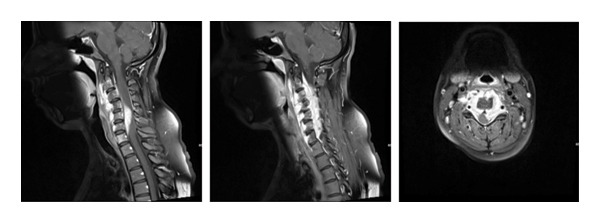
Enhanced cervical spine MRI: on postgadolinium T1WI, the epidural spinal mass showed avid enhancement with confirmation of extension into the prevertebral space and neural foramina bilaterally, causing cord compression from C2 to C7, predominantly on the left side. No collections were seen.

A soft collar was placed to protect the cervical spine. Despite the potential diagnosis of lymphoma, she was started on dexamethasone tablets (4 mg) every 6 h as a treatment for her cord compression symptoms. Four days after the steroid treatment, a CT‐guided biopsy was aborted because a preprocedural scan of the mass revealed a drastic improvement in the mass, compared to the imaging from 4 days prior, to the point where there was no longer enough of a safe margin to perform the biopsy without a potential risk of injury to the posterior pharyngeal/esophageal wall. The patient was kept on steroids after the procedure and awaited a repeat MRI scan; during this period, she reported notable improvement in her symptoms. A repeat cervical spine MRI (1 week after the initiation of dexamethasone) revealed a significant decrease in the size of the cervical lesion (Figure 2).

**FIGURE 2:**
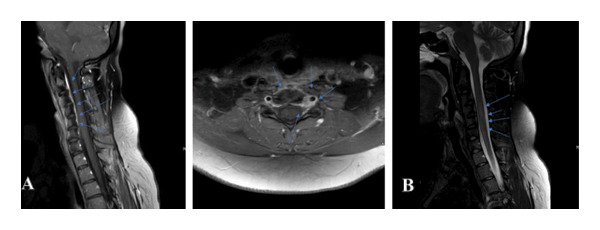
Cervical spine MRI after steroid treatment: (A) post‐GD, T1WI revealed significant improvement in enhancing epidural and prevertebral masses with small residuals. (B) On sag T2W images, there was significant improvement in spinal cord swelling and altered signals, with minimal residual altered signals.

The hematology service was consulted at this point, with their impression of the mass being most likely lymphoma. Upon their recommendation, the dexamethasone was discontinued as it can impair the diagnosis of lymphoma, and the MRI and positron emission tomography (PET) scans were repeated 2 weeks after the cessation of steroids.

Repeat MRI revealed interval progression of the cervical lesion, with associated compressive myelopathy, and PET/CT revealed a prominent fluorodeoxyglucose (FDG)‐avid intradural extramedullary cervical soft tissue mass with extraforaminal and prevertebral extension, with no other suspicious avid FDG activity anywhere else (Figure 3). A CT‐guided biopsy yielded a limited amount of lesional tissue. Pathology revealed a granuloma in a background of non‐neoplastic skeletal, muscular, and fibrous tissue, with negative staining for acid‐fast bacilli (AFB). The patient then underwent surgical biopsy, which revealed non‐necrotizing granulomatous lymphadenitis, with negative special stains for AFB and fungal elements. Immunohistochemistry confirmed the benign nature of the sample material, and *Mycobacterium TB* (MTB) PCR of the sample yielded negative results.

**FIGURE 3:**
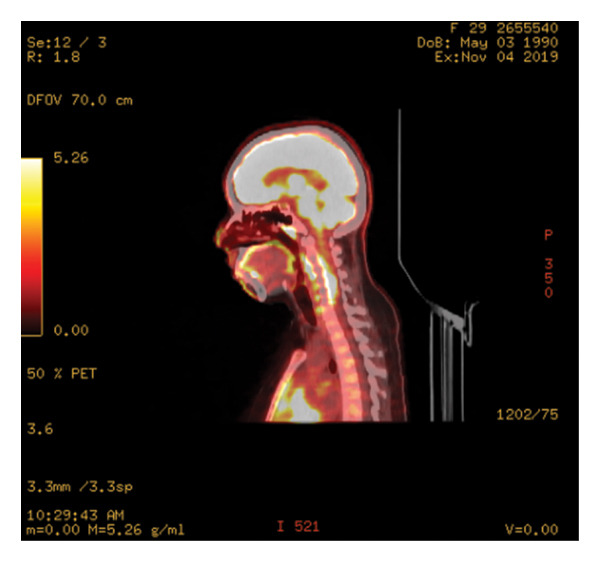
A PET‐CT scan utilizing an FDG‐18 radiotracer revealed avid tracer uptake from the cervical mass.

Following the biopsy result, the primary team called for a multidisciplinary meeting between rheumatology, infectious disease, and hematology. The consensus was possible neurosarcoidosis, and the planned treatment was steroids. Meanwhile, AFB cultures were followed for growth. The patient was started on prednisone 60 mg once daily with a tapering regimen. Two months postdischarge, she was seen in the neurology clinic. She was on a tapering dosage of steroids at the time and had experienced significant clinical improvement of the numbness and weakness. Repeat MRI revealed significant improvement of the mass, with mild residual tissue. The plan was to start rituximab on the basis of the clinical response to prednisolone and the lack of growth of AFB cultures after 4 weeks of incubation.

After more than 2 months of incubation, the AFB culture from the tissue taken from the open biopsy, which was inoculated on Lowenstein–Jensen (LJ) medium, was flagged as positive, indicating the growth of presumptive *M. riyadhense* identified using biochemical testing as well as MALDI‐TOF MS. The patient was lost to clinical follow‐up at that time, and subsequent attempts to contact her were unsuccessful. One year after her initial biopsy, she responded to a virtual clinic follow‐up call; at that time, she was clinically stable. Her symptoms of numbness and weakness had improved initially for the first 9 months after starting the steroids and rituximab. Still, they did not completely resolve, and she had begun to experience worsening symptoms over the past 2 months.

The patient was then started on a regimen of rifampin (600 mg/day), isoniazid (300 mg/day), pyrazinamide (1500 mg/day), ethambutol (1200 mg/day), and pyridoxine (40 mg/day). The sample from the surgical biopsy was sent to the Bioscientia Reference Laboratory (Ingelheim, Germany) for identification and susceptibility. Nucleic acid sequencing of 16S ribosomal RNA confirmed the isolate to be *M. riyadhense*. Susceptibility testing using the BACTEC MGIT system revealed that the isolate was sensitive to rifampin, clarithromycin, and azithromycin. The patient was maintained on the same regimen, with complete clinical resolution of her symptoms and almost complete resolution of the mass radiologically on follow‐up MRI (Figure 4). At her clinic appointment, the patient reported that she had stopped her therapy at 10 months, rather than complying with the initial planned duration of at least 1 year. A joint decision was made to observe her condition while off the therapy. At the virtual clinic follow‐up visit 1 year later, she remained asymptomatic, with no other new medical issues.

**FIGURE 4:**
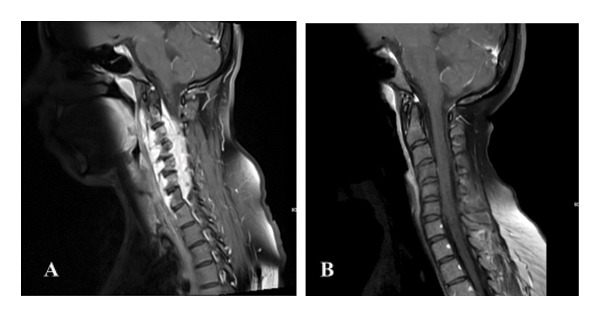
Cervical spine MRI posttreatment: the previously observed epidural and prevertebral enhancing masses were almost completely resolved, as were the altered spinal cord signals. No features of cord compression were observed. Note: the faint enhancement seen on the post‐GD T1W images (axial view) represents the normal enhancing dural coverings around the nerve roots′ exit. (A) Pretreatment T1WI with gadolinium, Oct 2019. (B) Posttreatment T1WI with gadolinium, Mar 2022.

## 3. Discussion

*M. riyadhense* is a slow‐growing NTM that can cause both pulmonary disease and extrapulmonary disease. It was initially discovered in 2009 in a young male patient who presented with persistent sinusitis after blunt trauma to the face [9]. Maxillary sinus lavage yielded mycobacteria with an unusual colony morphology that was initially misidentified as MTB by line probe assays. Further analysis via 23S rRNA sequencing led to the discovery of *M. riyadhense* [9]. Although sporadic cases have been identified in France, Bahrain, and South Korea, the majority of the identified cases have been reported from Saudi Arabia and the city of Riyadh [13].

The apparent predominance of *M. riyadhense* cases in Saudi Arabia likely reflects a complex interplay of several factors. NTM are ubiquitous environmental organisms, and species distribution varies considerably by geographic region, influenced by climate, water chemistry, and soil composition [14]. Saudi Arabia has unique environmental characteristics with extreme temperature variations, low humidity, and specific soil mineral compositions that differ markedly from other parts of the world. Similar geographic clustering has been observed with other NTM species; for example, *Mycobacterium ulcerans* (Buruli ulcer) has been historically largely confined to specific regions in Africa and Australia, and although it continues to grow worldwide, the old clusters in Australia and countries such as Nigeria have had a rapid increase in the rate and severity of cases [15]. As another example of NTM clustering, *Mycobacterium kansasii* initially showed a higher prevalence in certain areas of the United States and Europe, with newer clusters in East Asia, Africa, and Europe being recognized over the past decade, possibly linked to industrialization [16, 17].

Another contributing factor may be the detection bias of advanced molecular diagnostic technologies. Accurate identification requires advanced molecular techniques such as 16S rRNA gene sequencing and MALDI‐TOF, which have been adapted by several reference laboratories in Saudi Arabia. In contrast, commercial line probe assays, which are still utilized by many laboratories worldwide, can misidentify M. riyadhense as MTB due to genetic similarities, and these [9, 13, 18].

Following the initial description of *M. riyadhense* in 2009 [9], there has been heightened awareness among infectious disease specialists and microbiologists in Saudi Arabia regarding this pathogen [18]. While diagnostic and ascertainment biases likely contribute substantially to the observed geographic distribution, we cannot exclude the possibility that *M. riyadhense* is genuinely more prevalent in Saudi Arabia and neighboring Gulf states. Understanding the true environmental distribution of *M. riyadhense* would require systematic environmental sampling and molecular surveillance across different geographic regions, which has not been conducted to date.

From 2009 to 2025, 32 cases were identified: 22 cases with pulmonary infections, 4 cases with lymphadenitis, and 2 cases with CNS involvement. Both CNS cases involved young women presenting with a CNS mass [18]. The first case involved an 18‐year‐old female who presented with a 3‐year history of headache and a new‐onset seizure, with a right frontal mass found on imaging. She was treated with a 4‐drug MTB regimen, later modified based on susceptibility results to rifampin and ethambutol only, for approximately 15 months with good response. The second patient was a 24‐year‐old female who presented with spinal cord compression from sclerotic lesions and an extradural mass in the thoracic spine. She achieved complete clinical resolution on the standard 4‐drug MTB regimen for 6 months, followed by 7 additional months of rifampin and ethambutol [12].

Infections of the CNS by NTM are uncommon. In a recent review of NTM implicated in CNS infections between 1980 and 2022, 112 out of 153 cases were eligible for assessment. *Mycobacterium avium* complex was the most frequently isolated pathogen in 34.8% of the cases, followed by *Mycobacterium fortuitum*; intracranial abscesses (36.6%) and leptomeningeal enhancement (28%) were the most common neuroimaging findings, more than 50% of patients had predisposing risk factors (immunocompromised or previous neurosurgical procedures requiring hardware implantation) [19]. Not a single case of *M. riyadhense* was reported. Our case is the third reported case from Saudi Arabia implicating this pathogen in CNS infection in patients with no recognized immunodeficiency.

Our patient was initially treated for CNS sarcoidosis with steroids and one dose of rituximab. There was a partial improvement clinically and radiographically, which probably resulted from regression of the associated edema and cord compression. However, the patient’s symptoms recurred within a few weeks after initial partial improvement. Although the use of steroids is a well‐described risk factor for NTM infection, corticosteroids are also used as adjuncts in the treatment of MTB to limit the inflammatory response and treat the immune reconstitution response, which has rarely been reported for disseminated NTM infection [20, 21]. Immune modulations have variable effects on the course of certain infections. In patients with IFN‐gamma–IL‐12 axis defects caused by anti‐IFN‐gamma autoantibodies, anti‐CD20 agents have been shown to be effective in the treatment of disseminated NTM infection [22–25].

The diagnostic dilemma of TB and sarcoidosis remains a challenge in the absence of a definitive diagnostic method for sarcoidosis. The clinical and radiological characteristics of both diseases can overlap, and both conditions result in granulomatous changes [26, 27]. Evidence of both NTM and TB has been reported in several older cases of sarcoidosis [28–35], and concomitant diagnosis of both diseases continues to be reported in more recent reviews and case reports [36–40]. There are multiple examples of mycobacteria being isolated from sarcoidosis patients, especially in recent literature with better diagnostics, supporting a connection between the two pathologies [37, 38, 40]. Even when looking at older literature, when molecular and immunologic diagnostics were performed on samples, mycobacterial DNA and RNA are present in up to 48% of sarcoid biopsies, suggesting that patients with sarcoidosis not undergoing advanced molecular studies could have missed mycobacterial disease [41, 42].

The treatment of *M. riyadhense* poses a challenge because of the lack of clinical trials or guidelines to guide the choice of treatment. Regimens are based on a few clinical reports and often resemble the standard courses used in MTB treatment [10–12, 18, 43]. The choice of regimens is based, in part, on the similarity of *M. riyadhense* to MTB in genome sequencing [44]. The susceptibility data from our case demonstrate that *M. riyadhense* was sensitive to rifampin, clarithromycin, and azithromycin. This information is particularly valuable given the rarity of this pathogen and the absence of standardized treatment guidelines. Such susceptibility data can guide clinicians who cannot perform susceptibility testing themselves, are awaiting results, or are developing treatment protocols for this organism. Another value of susceptibility testing will be guidance of treatment choices when first‐line agents fail, or are contraindicated, due to adverse effects or drug‐drug interactions.

NTMs provide multiple diagnostic challenges due to the heterogeneity of their presentations and special requirements to grow the isolate, such as the prolonged incubation in our case. Physicians should be vigilant when sending clinical samples of tissue with inflammatory changes to include AFB testing, keep samples for longer incubation, and, in case of growth, to ensure isolates are properly identified and tested for susceptibility, which is often performed through reference laboratories. Since its initial identification in 2009, *M. riyadhense* has already been demonstrated to cause a wide variety of infections, from pulmonary infections to CNS masses, and should be included in the differential diagnosis of such presentations, especially in patients living in Saudi Arabia and the Gulf region. *M. riyadhense* seems to respond well to treatment courses that more closely resemble traditional regimens used for MTB, in both the choice of agents and shorter durations, compared to other NTMs.

## Funding

No funding was received for this study.

## Consent

No written consent has been obtained from the patient as there are no patient identifiable data included in this case report/series.

## Conflicts of Interest

The authors declare no conflicts of interest.

## Data Availability

The data that support the findings of this study are available on request from the corresponding author. The data are not publicly available due to privacy or ethical restrictions.
